# Rationale and Design of a Remote Web-Based Daily Diary Study Examining Sexual Minority Stress, Relationship Factors, and Alcohol Use in Same-Sex Female Couples Across the United States: Study Protocol of Project Relate

**DOI:** 10.2196/11718

**Published:** 2019-02-04

**Authors:** Kristin E Heron, Robin J Lewis, Alexander T Shappie, Charlotte A Dawson, Rachel Amerson, Abby L Braitman, Barbara A Winstead, Michelle L Kelley

**Affiliations:** 1 Department of Psychology Old Dominion University Norfolk, VA United States; 2 Virginia Consortium Program in Clinical Psychology Norfolk, VA United States

**Keywords:** sexual and gender minorities, ecological momentary assessment, alcohol drinking, family relations, stress, psychological

## Abstract

**Background:**

The Healthy People 2020 initiative aims to reduce health disparities, including alcohol use, among sexual minority women (SMW; eg, lesbian, bisexual, queer, and pansexual). Compared with heterosexual women, SMW engage in more hazardous drinking and report more alcohol-related problems. Sexual minority stress (ie, the unique experiences associated with stigmatization and marginalization) has been associated with alcohol use among SMW. Among heterosexuals, relationship factors (eg, partner violence and drinking apart vs together) have also been associated with alcohol use. Negative affect has also been identified as a contributor to alcohol use. To date, most studies examining alcohol use among SMW have used cross-sectional or longitudinal designs.

**Objective:**

Project Relate was designed to increase our understanding of alcohol use among young SMW who are at risk for alcohol problems. The primary objectives of this study are to identify daily factors, as well as potential person-level risk and protective factors, which may contribute to alcohol use in SMW. Secondary objectives include examining other physical and mental concerns in this sample (eg, other substance use, eating, physical activity, and stress).

**Methods:**

Both partners of a female same-sex couple (aged 18-35 years; n=150 couples) are being enrolled in the study following preliminary screening by a market research firm that specializes in recruiting sexual minority individuals. Web-based surveys are being used to collect information about the primary constructs of interest (daily experiences of alcohol use, sexual minority stress, relationship interactions, and mood) as well as secondary measures of other physical and mental health constructs. Data are collected entirely remotely from women across the United States. Each member of eligible couples completes a baseline survey and then 14 days of daily surveys each morning. Data will be analyzed using multilevel structural equation modeling.

**Results:**

To date, 208 women (ie, 104 couples) were successfully screened and enrolled into the study. In total, 164 women have completed the 14-day daily protocol. Compliance with completing the daily diaries has been excellent, with participants on average completing 92% of the daily diaries. Data collection will be completed in fall 2018, with results published as early as 2019 or 2020.

**Conclusions:**

Project Relate is designed to increase our understanding of between- and within-person processes underlying hazardous drinking in understudied, at-risk SMW. The study includes a remote daily diary methodology to provide insight into variables that may be associated with daily hazardous alcohol use. Before the development of programs that address hazardous alcohol use among young SMW, there is a need for better understanding of individual and dyadic variables that contribute to risk in this population. The unique challenges of recruiting and enrolling SMW from across the United States in a daily diary study are discussed.

**International Registered Report Identifier (IRRID):**

DERR1-10.2196/11718

## Introduction

### Background

One of the goals of Healthy People 2020 [1], a science-based report issued by the US Office of Disease Prevention and Health Promotion that identifies national objectives for improving the health of Americans, is to specifically improve the health and well-being of lesbian, gay, bisexual, and transgender (LGBT) individuals. Compared with heterosexual women, sexual minority women (SMW; eg, lesbian, bisexual, queer, and pansexual) engage in more heavy drinking and have lower rates of abstention from drinking [2-4]. SMW also experience more negative alcohol-related consequences than heterosexual women, such as driving under the influence of alcohol, having unplanned sex, having suicidal thoughts during or after drinking, fighting or arguing with someone, and engaging in sexual harassment during/after drinking [5,6]. Despite evidence for health disparities between heterosexual women and SMW in alcohol use, much remains to be learned regarding why some SMW in particular are at risk. The goal of this study, Project Relate, was to increase the understanding of hazardous drinking among SMW who are in same-sex relationships using an entirely remote Web-based data collection procedure, including a daily diary component. This approach permits examination of research questions at multiple levels, allowing us to consider between-couple characteristics (ie, couple-level; answering the question: *which couples* drink more), between-person characteristics (ie, person-level; answering the question: *who* drinks more), and within-person processes (ie, daily level; answering the question: *when* does one drink more). The primary objective of this study is to examine the daily associations between hazardous alcohol use and sexual minority stress, affect, and relationship factors among young same-sex female couples. The theoretical framework and existing empirical evidence that contributed to the development of this study are described below.

### Sexual Minority Stress and Alcohol Use

Members of minority groups experience unique stressors related to their stigmatized and marginalized social status in society. Stigmatization and marginalization based on sexual identity or orientation is known as sexual minority stress [7-9]. Specifically, Meyer’s [9] model of sexual minority stress involves experiences of harassment or discrimination related to sexual identity/sexual orientation (ie, heterosexism), the expectation of and vigilance toward these events, concealment of sexual identity, and the internalization of negative societal attitudes toward one’s sexual identity (eg, internalized heterosexism). Together, these experiences create additional stressors beyond those experienced by majority of individuals. There is ample evidence that sexual minority stress is associated with negative health behaviors, such as alcohol use, through emotion regulation and coping strategies [8,10-14] as well as through less perceived social support [12] and more social constraints and social isolation [13]. Although these studies provide important information regarding how minority stress is associated with negative affect and alcohol use at the between-person level, it is important to note that these studies used primarily cross sectional (ie, single assessment points) and traditional longitudinal designs (ie, using repeated assessments over weeks, months, or years). These types of designs limit our understanding of within-person processes by either studying processes as a between-person variable (ie, using cross-sectional designs) or by studying long-term within-person differences (ie, using traditional longitudinal designs). A handful of studies have used daily or momentary assessment of stress and alcohol use among sexual minorities. For example, in research of young men who have sex with men, alcohol use was greater on days when men indicated they were drinking to cope with stress [15] and on days when general stress was reported [16]. Furthermore, in a 9-day daily diary study, the interaction of structural stigma (eg, state policies that promote discrimination) and rejection sensitivity was associated with alcohol use among young men who have sex with men. Most relevant to this study was the finding that discrimination was associated with contemporaneous and prospective substance use in a group of sexual and gender minority individuals [17]. However, the question of how *daily* experiences of sexual minority stress and negative affect contribute to SMW’s hazardous drinking remains. In this study, we will test whether greater daily sexual minority stress is directly associated with more alcohol use and associated problems as well as examine indirect associations through negative affect.

### Relationship Factors and Alcohol Use

In addition to the link between sexual minority stressors, negative affect, and alcohol use, it is important to consider the associations between relationship factors and alcohol use. A review of studies on heterosexual couples concluded that unpartnered individuals drank more compared with those in serious romantic relationships and that drinking is associated with poorer relationship quality as well as conflict and violence [18]. Among SMW, several studies have demonstrated associations between partner violence and alcohol use and related problems in samples of SMW. For example, in a study of lesbian couples who experienced intimate partner violence, approximately 64% of both batterers and victims report using alcohol or drugs before the violent incidents [19]. Alcohol use was also associated with SMW’s nonphysical domestic violence (eg, verbal threats and damage to property) [20]. Research also suggests that intimate partner violence among SMW is associated with hazardous alcohol use [21], which may stem from emotional distress [22] or sexual minority stressors [23].

Another important characteristic when studying relationships and hazardous alcohol use is the degree to which partners drink together versus separately. Among heterosexuals, drinking together has been associated with more intimacy and fewer negative partner behaviors than drinking apart [24]. Conversely, drinking apart is associated with more negative relationship events [24] as well as less satisfaction, more conflict, and marital dissolution [18,25]. Among lesbian women, when controlling for physical and psychological aggression, discrepant drinking (ie, differences in partners’ drinking quantity) is associated with poorer relationship adjustment [26]. Furthermore, in a longitudinal examination of discrepant drinking and intimate partner violence among lesbian partners, a cyclical pattern of alcohol use and violence occurred such that discrepant drinking predicted psychological aggression 6 months later, which in turn predicted later discrepant drinking. Physical violence also predicted subsequent discrepant drinking 6 months later [27]. Considering the small body of work connecting alcohol use to relationship variables in SMW, in this study, we will examine how instances of partners drinking together versus apart and discrepant drinking are associated with relationship functioning on the same day and subsequent days and in turn, how relationship functioning may then be associated with drinking on subsequent days.

### Protective Factors

The impact of minority stress and negative relationship factors may be modifiable at the couple- or individual-level [9,28], but more information is needed to understand what factors may protect SMW from problematic alcohol use [29]. Therefore, the final aim of this research is to investigate societal-, couple-, and person-level characteristics that could be associated with daily experiences of sexual minority stress, relationship quality, and drinking or that may strengthen or weaken the associations among them. For example, connection to the sexual minority community, a stronger sexual minority identity, and social support may all protect against the deleterious effects of sexual minority stress and related negative mental and behavioral outcomes [30-32]. Similarly, being in a legally recognized relationship may also be a protective factor against sexual minority stress and negative health outcomes [33-35]. Although marriage between same-sex individuals is now legal in all states in the United States, other protections (eg, housing, employment, and health care benefits) are not federally mandated and, thus, vary by state. The lack of these state protections (ie, structural stigma) has been associated with health outcomes. For example, in a study published in 2014, sexual minority individuals who lived in states without protections for sexual minorities (eg, in housing and employment benefits) and that banned same sex marriage had a higher prevalence of psychiatric disorders than those who lived in states with such protections [36]. In this study, we will assess a variety of potential factors that may protect against hazardous drinking, including both factors specific to the person or couple (eg, connection to sexual minority community and being in a committed relationship), as well as structural factors (eg, living in a state with protections for people in same-sex relationships). Identification of potentially modifiable individual risk and protective factors can facilitate development of culturally tailored inventions to reduce hazardous drinking. Furthermore, identification of structural (societal) risk and protective factors may suggest important directions for advocacy efforts.

### Study Objectives

The primary objective of Project Relate is to increase our understanding of hazardous drinking among SMW. Using a daily diary methodology, this project investigates the associations among sexual minority stress, affect, relationship factors, and alcohol use. The development of this study was guided by minority stress theories [7-9] that offer a way to understand the unique stressors that SMW experience related to their marginalized societal status. In addition, because relationship factors are important correlates of alcohol use among heterosexual couples [18], we consider how relationship characteristics are associated with alcohol use among young adult same-sex female couples. In addition, we also look at potential protective factors. Collecting information on sexual minority stressors, emotional functioning, relationship experiences, and alcohol use from both partners will allow us to assess the interactions among these variables as they enhance or reduce the quality of life of SMW. Extensive background information, including personality characteristics, social support, history of discrimination, and community context, will provide information on personal and contextual factors that moderate these associations. A secondary objective of Project Relate is to examine other physical and mental health concerns, including other substance use, eating, physical activity, and general mental health and well-being among SMW. The opportunity to examine daily-, individual-, and couple-level effects is a particular strength of the study methodology.

## Methods

### Project Overview

Project Relate is an ongoing daily diary study of same-sex female couples. Young adult SMW (aged 18-35 years) are the target sample for this study because in community samples they frequently endorse indicators of hazardous drinking [3]. Both partners of the romantic relationship are enrolled to capture information about the relationship from both members’ perspectives. Participants are recruited from across the United States, and data collection is occurring remotely via Web-based surveys. Both women in each couple complete a comprehensive baseline survey and then a brief daily survey each morning for the following 14 days. Each member of the couple begins the daily survey on the same day to ensure we have corresponding daily data for each couple. The Old Dominion University Institutional Review Board approved all study procedures (Project #839097).

### Power Analysis

A power analysis to determine the sample size needed for planned analyses was conducted in 2 steps. First, a power analysis was conducted for traditional structural equation modeling using Monte Carlo simulation methods [37], focusing on powering the hardest effects to detect (the small indirect effects, beta=.10). Similar models were conducted incorporating a small-to-medium effect–moderating variable (beta=.20). Monte Carlo simulation methods indicated that for the effect size expected, a sample size of 200 *cases* yields sufficient power (0.808) to detect the relevant effects. This, however, assumes no correlation among residuals, which we know is not true in nested designs. To determine how to appropriately account for the correlated observations and identify the minimum number of participants needed for power of .8 in the multilevel design, a formula taking into account the expected intraclass correlation coefficients (ICCs), or degree of relatedness within couples, for key variables was used [38]. Assuming an average of 11 days of assessments (80% response rate to account for attrition) and 150 couples, an ICC up to approximately .70 would yield the necessary number of *cases* to maintain a power of .80. Previous daily diary alcohol research suggests we can expect to see lower ICCs, likely ranging from .4 to .7 [39], yielding more power. Therefore, recruiting 150 couples (N=300 people) is more than adequate to provide sufficient power.

### Participant Selection

Recruiting large samples of sexual minorities is difficult to do in many localities, and therefore, we are recruiting participants from across the United States. We partnered with Community Marketing and Insights (CMI), a leading market research firm specializing in Web-based research with the LGBT community. CMI maintains a proprietary research-only panel of over 90,000 individuals who identify as sexual minorities and regularly take part in market research and health research (although psychological research studies are less common). CMI recruits participants to take part in this study from their existing panel and potential new panel members who may be interested in participating in research studies. CMI is consistently recruiting new research panel members through LGBT print, digital, and event outreach activities as well as LGBT-specific outreach on social media sites (eg, Facebook, Craigslist, and sexual minority-specific social media).

To be eligible for the study, both partners have to meet the following eligibility criteria: (1) aged 18 to 35 years, (2) self-identify as a cisgender woman (meaning self-identify as a woman and was assigned female at birth, ie, she is not transgender), (3) currently in a romantic relationship with a woman for at least 3 months, (4) see partner in person at least once per week, and (5) able to respond to daily surveys between 6 am and 12 pm for 2 weeks. In addition to these 5 criteria that must be met by all participants, at least one person in the couple must also meet the following 3 criteria: (6) only or mostly attracted to women, (7) drank alcohol at least 3 days in the previous 2 weeks, (8) drank 4 or more standard alcoholic drinks in 1 sitting at least once in the previous 2 weeks (ie, met criteria for a binge drinking episode).

These inclusion criteria were developed for several reasons. First, although we recognize that people with other gender identities (eg, transgender and nonbinary gender) and sexual identities (eg, bisexual women not in a relationship with a woman) are also at risk for experiencing both sexual minority stress and hazardous alcohol use, individuals with different gender and sexual identities likely experience stressors that differ from one another [29]. In other words, a transgender and cisgender woman’s or a lesbian and bisexual woman’s daily experiences might differ from each other. Thus, this study focuses solely on individuals who identify as cisgender female and are in a relationship with a woman. Second, we require the couples to see each other in-person at least once a week to avoid potential confounds related to being in a long-distance relationship. Third, although 1 participant has to report only or mostly attracted to women, her partner can describe her attraction in other ways (eg, attracted to men and women equally); this criterion was used both to obtain a sample size large enough to test study aims and to have some variability in attraction (eg, attracted to men and women) and identity (eg, lesbian, bisexual, and queer) that can be explored statistically. Finally, the alcohol inclusion criteria for 1 participant is used to identify participants belonging to the desired at-risk alcohol use population of interest and to increase the likelihood of enrolling couples in which at least 1 partner drinks with some frequency, resulting in occurrence of drinking during the 14-day daily diary reporting period.

### Study Procedures

#### Recruitment

CMI initiates the recruitment process by contacting potential participants via email and providing them with a link to a Web-based screening survey. The potential participant is also asked if she thinks her partner would be interested in participating, and if so, to provide her email address. CMI then screens her partner for interest and eligibility, and once potentially eligible couples are identified, CMI provides the researchers with the email addresses of potential participants and their partners. Upon receiving contact information for potentially eligible couples, the researchers conduct a second set of screening assessments (using the criteria described above) to ensure eligibility of the couple.

#### Study Description and Informed Consent

Once eligibility and interest are verified for both people in the couple, they are emailed separately with introductory information about the study. Given that data collection is occurring entirely remotely, and daily assessment procedures are likely new to these participants, we developed detailed written and video materials describing the study purpose, procedures, risks, and benefits. The professionally developed videos consist of 5 brief (1-3 min) videos of the study investigators and research assistants describing the study. Corresponding written materials were also developed. Given that developing these videos took additional time and resources above what was required to develop the written materials, we built in a design feature to assess the video utility. Specifically, couples were randomized in blocks of 6 to 1 of 2 introductory information groups: (1) videos plus written materials (video+written) or (2) written materials only (written-only). Couples are randomized (instead of individual participants) to limit contamination between partners. Those in the video+written group receive a Qualtrics survey Web link that guides participants through the series of 5 videos explaining the purpose and procedures of the study; the written material is also available below the video. Couples assigned to the written-only group receive a similar Qualtrics survey link where they review the same information presented in the videos, but in text form only. The written materials provided for participants can be found in Multimedia Appendix 1. The length of time participants spend reviewing the materials (including time spent on each individual video and corresponding written text) is automatically tracked. Immediately after reviewing the study information, participants are presented with the informed consent document. If participants do not consent within 2 to 3 days of the initial email, they are sent 2 additional email reminders (approximately 2-3 days apart) regarding the study and consent procedures. Both women in the couple must provide consent before either can begin the study.

#### Baseline Survey

After both members of the couple provide consent, the couple is enrolled in the study. At that time, each participant receives an email via Qualtrics with an individual link to her baseline survey. The survey takes approximately 30 min to complete. In the email, participants are asked to complete the baseline survey within 3 days. Similar to the consent procedures, if participants do not complete the baseline survey within 2 to 3 days, 2 email reminders are sent approximately 2 to 3 days apart. Both members of the couple must complete the baseline survey to move on to the daily survey. If only 1 partner completes the baseline survey and her partner does not, she is thanked, compensated for completing the baseline survey, and informed that her participation is complete (without being told her partner did not complete the baseline survey).

#### Daily Surveys

Once both members of the couple complete the baseline survey, they are notified separately via email of the starting date of the daily survey (which ideally occurs the day after they complete the baseline survey). Each person in the couple is sent separate automated emails through the Qualtrics survey system, with an individual link to their brief daily survey each morning at 6:00 am for the following 14 days. We selected a 14-day assessment period based on research demonstrating daily sexual minority stressors [40,41] and alcohol use [17,42] occur over this length of time as well as our screening criteria requiring binge drinking (in at least 1 partner) during the previous 2 weeks. Thus, using a 14-day daily assessment period should appropriately balance both participants’ burdens while ensuring the study is of sufficient duration to capture the experiences and behaviors of interest in daily life. Each daily survey takes approximately 5 min to complete. Participants are instructed to complete the survey independently from their partner using their own personal computer, tablet, or mobile device by 12:00 pm each day. The participants receive a reminder email after 2 consecutive days of missing or incomplete surveys.

Consistent with other daily diary studies of alcohol use [43,44], we elected to have participants complete surveys each morning (about the previous day) instead of at the end of the day (about that day) because our primary research questions of interest were regarding alcohol use, and end of day surveys could present 3 concerns. First, we were concerned we could *miss* reports of drinking if participants elected to complete the survey in the evening before drinking (or before they finished drinking). Second, some of our questions were regarding alcohol use consequences, and some consequences (eg, being hungover) may not emerge until many hours after drinking stops. Third, on days when participants were drinking, if we asked them to complete a survey in the evening, we were concerned they could be intoxicated while completing the daily survey. We realize that by asking participants to report on their previous day each morning (vs current day each evening) requires some additional retrospection; however, given the focus on alcohol use in this study, any concerns regarding additional retrospective bias were outweighed by the alcohol-related concerns noted above.

#### End of Study Survey

On the day after the final daily survey, participants receive an email with a link to take a survey specifically designed for this study about their reactions as a study participant. Additional details regarding this measure can be found in the Measures section.

#### Compensation

Participants and their partners each receive US $25 for completing the baseline survey and US $3 each for each daily survey, with a US $10 bonus for completing at least 80% of the daily surveys (ie, more than 11 days). Thus, each participant can earn a maximum of US $77 via their choice of check or gift card.

### Measures

All measures included in the baseline survey are described in Table 1. The measures are organized by those designed to test the primary study aims described above and secondary measures that will supplement testing of the primary aims. For each measure, we provide a brief description of the construct assessed. In addition, several modifications to measures were made and are worth noting. First, most measures in the baseline survey referencing a specific time frame were adapted to 3 months to be consistent throughout the survey and ensure we could examine these constructs during similar time periods. The time frames of the following measures were *not* adapted: the Conflict Tactics Scale [45], the Psychological Maltreatment of Women Inventory [46], the physical health questions Short Form-20 [47], the Daily Heterosexist Experience Questionnaire [48], and the Modified Eating Disorder Examination Questionnaire [49,50]. These measures were not changed to a 3-month time frame because their original time frames (eg, last year and last month) were more appropriate for addressing the study aims. Second, changes were made to several of the measures to be appropriate for same-sex female couples. These adaptions included changing male or gender-neutral pronouns to female pronouns and modifying sexual behavior items that require male partners. Finally, because of the length of the baseline survey, attention check items were added to ensure that participants are paying attention and selecting their answers carefully. Attention check items are included as items in some of the longer measures as well as stand-alone questions. Rationale for inclusion of these items is discussed in more detail in the Discussion section below.

**Table 1:** Baseline measures.

Construct and description			Measure name
**Demographic information**			
	Person	Age, ethnicity, race, sexual orientation (identity, attraction, and behavior), gender, height, and weight	Developed for this study
	Relationship	Status, length, and living situation	Developed for this study
	Context	Employment status, education, income, and residence	Developed for this study
**Primary measures**			
	Relationship functioning	Psychological and physical violence toward or from a partner	Conflict Tactics Scale (CTS2)—physical assault and sexual coercion subscales (modified to be appropriate for same-sex relationships) [45]
		Maltreatment in an intimate relationship (emotional/verbal and domination/isolation)	Psychological Maltreatment of Women Inventory-short version [46]
		Satisfaction with the relationship and perceptions about partners’ satisfaction, commitment, and security with the relationship	No scale name [51]
		Report of partners’ behavior (hostile, jealous, and emotionally supportive)	Reports of partners’ behavior [51]
		Commitment to romantic relationship	Commitment subscale from the Investment Model [52]
		Feelings of jealousy toward romantic partner	Multidimensional Jealousy Scale- cognitive subscale [53]
		Anxious expectations of potential rejection from others, generally	Adult Rejection Sensitivity Questionnaire [54]
	Alcohol use	Identification of at-risk drinkers (hazardous alcohol use, dependence symptoms, and harmful alcohol use)	Alcohol Use Disorders Identification Test [55]
		Typical weekly alcohol consumption	Daily Drinking Questionnaire [56]
		Importance of certain reasons in someone’s decision not to drink	Reasons for Not Drinking [57]
		Alcohol consequences experienced by young adult drinkers	Brief Young Adult Alcohol Consequences Questionnaire [58]
		Drinking behavior related to partner interaction	Partner drinking questions [59]
		Drinking motives (social, coping, enhancement, and conformity)	Drinking Motives Questionnaire [60]
	Sexual minority stress	Sexual minority identity and psychosocial functioning	Lesbian, Gay, Bisexual Identity Scale [61]
		Openness about sexual identity and orientation	Single item openness question [62]
		Anxious expectations of potential rejection from others as a result of sexual minority identity	Sexual Minority Women Rejection Sensitivity [63]
		Experiences of sexual stigma	Daily Heterosexist Experiences Questionnaire—all subscales except parenting and HIV/AIDS [48]
	Social support and resilience	Support from family, friends, and significant others	Multidimensional Scale of Perceived Social Support [64]
		Ability to recover from stress or “bounce back”	Brief Resilience Scale [65]
**Secondary measures**			
	Physical health	General health, bodily pain, and activity limitations	20-Item Short Form Survey Instrument [47]
	Mental health	Aggression (physical aggression, verbal aggression, anger, and hostility)	Buss-Perry Aggression Questionnaire Short Form [66]
		Psychological distress and well-being (anxiety, depression, behavioral control, and positive affect)	Mental Health Inventory [67]
		Suicidal behaviors and thoughts	Inventory of Depression and Anxiety Symptoms- Suicidality Subscale [68]
	Family	Exposure to interparental violence	Items adapted from CTS2 [45]
	Drug use	History of marijuana use and form of ingestion	Marijuana use [69]
	Disordered eating	Eating pathology (body dissatisfaction, binge eating, cognitive restraint, purging, restricting, and excessive exercise)	Eating Pathology Symptoms Inventory [70,71]
		Diagnostic criteria for eating disorder behaviors	Modified Eating Disorder Examination Questionnaire [49,50]

The daily survey measures are described in Table 2 and are similarly organized by primary and secondary constructs of interest. Most of the primary and secondary measures included in the daily survey were adapted from existing daily diary or ecological momentary assessment (EMA) studies or were developed for this study to assess daily behaviors, particularly drinking behaviors and sexual minority stressors. Of note, when adapting items from existing scales, for example, to assess daily sexual minority stressors, we selected and edited items to be appropriate for a daily time frame (ie, would vary day-to-day). To balance the survey length on days when a participant did not drink or interact with her partner, filler items regarding media use, time management, and general social interactions were developed and are administered on nondrinking and noninteraction days. Finally, the format of the survey was optimized for mobile delivery. For example, visual analog slider scales are used instead of matrix tables in some cases so that all response options are visible to the participant when using a smaller screen (eg, on a smartphone). Additional details regarding the daily survey measures can be found in Table 2, and all daily survey items can be found in Multimedia Appendix 2.

The end-of-study survey contains questions concerning whether the participant believed her experiences were captured by the questions asked (eg, “While answering questions related to your gender and/or sexual identity, did you think that the questions you answered were inclusive of the way you describe yourself?”) as well as what was her preferred method of accessing the survey materials (ie, computer, tablet, or smartphone). Participants are also asked about their motivation to improve their health and whether they would be comfortable using mobile technology to monitor their physical and mental health. Survey items are included in Multimedia Appendix 3. The feedback gathered from the end-of-study survey will be used to gauge the participant experience in this study as well as provide an opportunity to improve future studies and survey materials.

### Data Analysis Plan

The data being collected in this study are inherently multilevel in nature, with days (level 1) nested within individuals (level 2) nested within couples (level 3). In addition, our research questions incorporate aspects of mediation and moderated mediation. As such, we will utilize an approach that combines multilevel modeling (MLM) [86,87] with mediation pathways available through structural equation modeling. This combined approach, multilevel structural equation modeling (MSEM) [88], allows for bootstrapping of the models, which can accommodate non-normal underlying distributions, particularly relevant for alcohol use outcomes and indirect effects [89,90] such as those incorporated in our proposed models. Regarding missing data, like MLM, MSEM is robust to missing data (eg, skipped observations). In addition, analyses will be conducted in Mplus [91] using maximum likelihood estimation, which allows for the estimation of parameters using all available cases (ie, no listwise deletion for missing variables within a time point). Participants with complete data will be compared with participants with missing data to identify potential attrition biases, and significant predictors will be included as covariates in all subsequent analyses. The MSEM approach allows for the simultaneous examination of level 1 (daily variation), level 2 (person-specific levels), and level 3 (couple factor) associations among variables, including moderating effects that strengthen or weaken those associations.

**Table 2:** Daily measures.

Construct		Description	Measure name and/or reference
**Primary measures**			
	Daily relationship functioning	Relationship quality and satisfaction	Items adapted from the Daily Positive Relationship Quality Composite [72,73]
		Intimacy and conflict	Items adapted [74]
		Positive and negative partner behaviors	Items adapted from the Interpersonal Qualities Scale [75]
		Partner aggression	Items adapted [76]
	Daily alcohol use	Quantity, duration, interactions, and location	Items developed for the study to assess daily drinking behaviors
		Daily alcohol consequences	Items adapted from the Brief Young Adult Consequences Questionnaire for daily administration [77]
		Drinking motives (social, coping, enhancement, and conformity)	Items adapted from the Drinking Motives Questionnaire—social, enhancement, and conformity were combined to a single item per factor [60]
		Nondrinking questions	Items developed for the study to balance drinking questions
		Daily-level reasons for not drinking	Items adapted for the daily level from the Reasons for Not Drinking [78]
		Drinking intentions	Item developed for the study to assess drinking likelihood in the next 24 hours
		Partner drinking	Items developed for the study to assess partner drinking
	Daily sexual minority stressors	Daily sexual minority stressors	Lesbian Women’s Daily Sexual Minority Stressors Scale [41]
		Sexual minority discrimination	Items adapted from the Heterosexist Harassment, Rejection, and Discrimination Scale [79]
		Sexual identity concealment	Items adapted from the Nebraska Outness Scale [80]
	Affect	Positive and negative affect	Items adapted from different versions of the Positive and Negative Affect Schedule
**Secondary measures**			
	Drug use	Smoking and marijuana use	Items adapted [69]
	Eating and body image	Disordered eating	Items adapted from the Eating Attitudes Test [81] and the Eating Disorder Examination Questionnaire [49,50]
		Body dissatisfaction	Items developed to assess current satisfaction with body
	General stress and coping	Stressful or unpleasant experiences	Items adapted from the Daily Inventory of Stressful Events [82]
	Physical activity	Sitting, walking, moderate activities, and vigorous activities	Items adapted from the International Physical Activity Questionnaire [83]
	About the survey	Location of survey completion, type of device used, and perceived privacy	Items developed for the study to improve future research
**Filler items**			
	General social interactions	Importance and pleasantness of social interactions	Items developed for the study to balance questions about partner interactions
		Positive social exchanges	Items adapted from the Daily Social Exchanges Checklist [84]
	Media use	Television, internet, and social media use	Items developed for the study to balance drinking questions
	Time management	Short-range planning and time attitudes	Items adapted from the Time-Management Questionnaire [85]

## Results

To date, 228 women (114 couples) have been successfully screened and been identified as eligible to participate in the study. Of these eligible women, 208 women (104 couples) have consented to participate and, thus, have been enrolled in the study. During the consent process, to date we have had to send reminder emails about consenting to 26% of women (59/228); 49 of these 59 women (83%) completed the consent within several days of the email reminder. A total of 6 participants discontinued participation after beginning the study but before the 14-day daily diary surveys. Of the remaining 202 participants, 44 are actively completing the daily surveys, and 164 have completed the study. Of those participants who have completed the study, compliance with the daily diaries has been excellent; on average, participants have completed 92% of the surveys (or 13 of 14 days). In reviewing the distribution of compliance rates by participants, nearly all participants (97.6%, 160/164) have completed at least half of the daily surveys. Data collection and enrollment is ongoing and expected to conclude in fall 2018.

## Discussion

Drawing from the literatures on sexual minority specific processes (eg, sexual minority stress) and general processes (negative affect and relationship factors), the primary objective of Project Relate is to increase our understanding of between- and within-person processes underlying hazardous drinking in the understudied, at-risk population of SMW. A daily diary methodology is being used to gain insight into the affective, behavioral, and relationship experiences of female same-sex couples working toward the goal of reducing health disparities and improving the health of SMW.

### Methodological Challenges and Considerations

Recruitment of couples into research endeavors is always a challenge, and recruiting same-sex couples creates additional challenges. Consequently, we devoted substantial effort to develop and refine processes for participant recruitment and retention. Below, we discuss the challenges faced when designing and implementing Project Relate.

#### Participant Recruitment Decisions and Issues

Several challenges emerged when designing and implementing this study regarding identifying and enrolling participants. First, working with a market research firm to recruit has advantages but also presents challenges. CMI has a large SMW panel who regularly complete Web-based surveys and access to other recruitment resources. The CMI employees are knowledgeable about best practices for Web-based recruitment and survey completion and contribute important ideas to assist with recruitment and retention of participants (eg, length of survey, compensation, and phrasing). At the same time, because CMI does relatively less social science research (compared with traditional market research for products), it was necessary to figure out how to “speak the same language” so that we could be faithful to the best social science practices (eg, measurement fidelity and human subjects’ protections). For this study, CMI is recruiting both through their proprietary panel of individuals who identify as LGBT and through digital and event outreach activities, including on social media sites such as Facebook, Craigslist, and sexual minority-specific sites. By using multiple recruitment sources, it is our goal to recruit a sample of SMW who are diverse in terms of their race/ethnicity and in other ways (eg, education and socioeconomic status). However, it is already a challenge to recruit young sexual minorities for research studies, and therefore, we recognize that to recruit a sufficient sample, it is possible that our sample will be limited in demographic characteristics.

Second, early in the study recruitment process, we observed a trend in the way in which young SMW label their sexual identity, which seemed to suggest that they were not identifying as lesbian as frequently as we had expected. In consultation with CMI, we learned that younger SMW whose attraction and behavior are consistent with the traditional definition of the “lesbian” label (ie, attracted to women or romantically involved with women) are either choosing to identify in other ways (eg, queer or pansexual) or are resisting labels altogether. Our initial planned inclusion criteria required at least 1 partner to identify as lesbian, but as a result of this consultation with CMI as well as internal discussions among our investigators, within the first month of recruitment, we changed this inclusionary criterion to “only or mostly attracted to women” described in the Methods section above. As researchers, our drive toward internal validity encourages us to recruit a homogeneous sample of 1 type of SMW (ie, lesbian women) that we can describe clearly; however, this group may not accurately represent the larger population of SMW, which is quite heterogeneous. This issue of how to appropriately define and describe sexual minority individuals is not new [92-94] and will likely continue to be a challenge for researchers as labels evolve over time and is something we will continue to attend to when designing future studies in this line of work.

Third, given that we are recruiting couples, we were very intentional in our protocol design regarding how and when we contacted participants to ensure privacy, confidentiality, and data fidelity. For example, when communicating with participants, we always contact individuals, never both people in the couple together (eg, in the same email). It is important that no participant feels coerced to participate because of her partner’s interest in the project. However, given this is a study of couples, both partners have to consent and complete the baseline survey before continuing in the study and must begin the daily diaries on the same day. In the rare instances when 1 partner does not wish to continue, we thank the other partner for her participation and compensate her for the portions of the study she completed but do not mention her partner’s noncompliance. In addition, to try to ensure we are getting independent responses from each participant, in all of our enrollment materials, we instructed participants not to complete the surveys together; we send them individualized survey links daily, and at the end of the daily survey, we enquire about the level of privacy each individual had when completing the survey. These privacy and data fidelity challenges are not unique to studies with sexual minorities but are experienced by researchers studying couples, and the concerns about collecting independent reports are likely more apparent in daily diary or EMA studies because of the frequent, repeated assessments in daily life.

#### Informational and Training Videos

To our knowledge, this study is one of the first to recruit same-sex couples for a completely remote Web-based daily diary collections, and thus, we decided to develop a series of videos to explain the research. To evaluate the efficacy of these videos, couples were randomized to either a written-only or a video+written group to examine whether the videos improve recruitment, attrition, and/or compliance. The videos are considered part of the informed consent process and, thus, are presented before the consent form, allowing us to examine whether consent rates, study attrition, and compliance with the study protocol differ by group. Evaluating the added utility (or lack thereof) of the video instructions for participants in this way can help to inform the design of future daily diary or EMA studies that use remote data collection methods with young adult participants.

#### Inclusion of Attention Check Items

As this project relies completely on remote Web-based data collection, we were concerned that for the longer baseline survey, participants could begin to respond randomly, without fully reading each question. To attempt to identify if this is occurring, in the baseline survey, we included a series of attention check items, sometimes called “instructional manipulation checks” [95]. These are items embedded in the larger survey with instructions to select a particular answer (eg, “Please choose ‘Frequently’”) or questions that have factual answers (eg, “Which of the following is the largest number?”) and are similar in format to other items in the survey. The attention checks are only included in the baseline survey, given its length (approximately 30 min). The daily surveys are much shorter (approximately 5 min), so we are less concerned about maintaining attention, and instead worried that including attention check items every day could be excessively burdensome and irritating for participants.

Research shows that including participants who fail attention checks leads to reduced power to detect study aims [95]. Although some have questioned if the inclusion of attention checks can adversely affect scale validity, recent studies have shown that they do not [96,97], and in fact, the inclusion of attention checks can improve performance on subsequent survey items [98]. For this study, we are not interested in eliminating participants who are inattentive but rather helping them to become attentive. Thus, we introduce to participants in the survey instructions (and videos) that these attention checks are embedded to help maintain their attention. Moreover, after failing an attention check, the survey automatically provides feedback stating that we detected the participant was not fully reading the instructions and asking her to respond again. This feedback is repeated as necessary until she answers correctly. This type of real-time feedback on attention checks has been shown to improve subsequent performance [95].

### Future Research Areas

EMA studies of health behaviors [99], as well as EMA studies conducted in the context of couples or families (eg, in which more than one person in a couple/family reports EMA data) [100], are rapidly increasing in popularity because they can examine more nuanced associations that consider individual, dyadic, and contextual variables that may influence behavior within people and couples over time. Despite growing interest in daily health factors and related stressors such as minority stress and alcohol use, research on daily experiences of sexual minority individuals lags far behind [101]. There are 2 important challenges that researchers are facing when conducting this work. First, as has been discussed at length elsewhere with respect to health behavior theories [102], existing theories may need to be refined or redeveloped to be able to guide our understanding of within-person processes that occur over relatively short time frames (eg, minutes, days, and weeks). Minority stress theories, including those that guided the development of Project Relate [8,9] at times, suggest predictions that involve within-person processes that may occur over short time frames but have largely been tested using cross-sectional designs (testing between-person processes) or traditional longitudinal designs (testing longer-term within-person processes). Project Relate is designed such that it will be able to test aspects of minority stress theories and presents an opportunity to extend and refine these theories to further the understanding of within-person processes in daily life.

A second challenge we encountered when designing Project Relate is the relative lack of well-established, validated measures that are appropriate for daily assessments. In Table 2, we provide a description of where we adapted measures from, based on either previous daily diary research with heterosexual couples or from existing retrospective measures. Importantly for Project Relate, we were unable to identify a measure of sexual minority stress that was appropriate for daily administration and, thus, created a daily sexual minority stressor measure [41] to use in this study. The lack of validated measures that are appropriate for daily or EMA studies highlights the importance for future work in this area to create new measures.

At present, the ability to provide education, mental health counseling, and interventions that are optimally effective for SMW is limited by our lack of understanding of key variables to incorporate in these efforts. Furthermore, the limited data available about relationship factors and alcohol use among SMW are typically derived from 1 respondent who provides information about herself and her partner. To address this limitation, our approach requiring both partners to participate is generating higher quality data on both partners’ affective, behavioral, and relationship experiences. In addition, we are including other variables both specific to SMW and heterosexual couples that may impact hazardous alcohol use. We believe it is critical to consider how couple-level dynamics/variables may impact hazardous alcohol use and related issues. In addition, we believe that understanding both daily and between-subjects’ mechanisms that may increase risk for hazardous alcohol is critical as is identifying protective factors that may attenuate this risk. By testing an empirically informed model that articulates probable within- and between-person factors and relationship variables that increase hazardous alcohol use, we believe this work will provide a necessary intermediary step toward informing culturally tailored prevention and intervention studies for SMW and contribute to a goal of the Healthy People 2020 initiative to improve the health and well-being of SMW.

## Appendix

Multimedia Appendix 1 Project Relate study introduction information for participants.

Multimedia Appendix 2 Daily diary survey items.

Multimedia Appendix 3 End of study survey items.

## Abbreviations

CMI: Community Marketing and Insights
EMA: ecological momentary assessment
ICCs: intraclass correlation coefficients
LGBT: lesbian, gay, bisexual, and transgender
MLM: multilevel modeling
MSEM: multilevel structural equation modeling
SMW: sexual minority women
