# Supplementary material for: Rationale and Design of a Remote Web-Based Daily Diary Study Examining Sexual Minority Stress, Relationship Factors, and Alcohol Use in Same-Sex Female Couples Across the United States: Study Protocol of Project Relate
Source: JMIR Res Protoc. 2019 Feb 4;8(2):e11718. doi: 10.2196/11718 (PMC6378553; doi:10.2196/11718)
Supplement: Multimedia Appendix 1 [file resprot_v8i2e11718_app1.pdf]

Appendix A

Project Relate Study Introduction Information for Participants

Page 1

Thank you for your interest in Project Relate!

On the next several pages we will give you some more information about the study, so you can decide if you are interested in participating.

Page 2

About This Study

This study is called Project Relate and the purpose is to learn more about sexual minority women's relationships and health. We know that young sexual minority women experience more health problems compared to heterosexual women, but we don't have a good understanding of why these differences occur, and that's something we are hoping to learn more about in this study.

Project Relate is different from other studies in two important ways.

1. **You will complete surveys daily.** We are very interested in learning more about the things that go on in your daily life. So, in order to get information about your day-to-day experiences, we'll ask you to fill out a brief web survey each morning for two weeks. The way we look at is that you are the expert on you and your life, and in this study we are hoping to give you a chance to tell us about some of the experiences, thoughts, and feelings you have each day.

2. **Both you and your partner will participate.** We are going to ask both you and your partner to fill out surveys. We know a fair amount about how the experiences of heterosexual couples are related to their health, but we know very little about same-sex female couples. It's our hope that by collecting information from both you and your partner that we will be able to improve our understanding of same-sex relationships and health.

Our ultimate goal with this research is to improve the health and well-being of young sexual minority women. We hope you'll consider participating in this study to help us work toward this important goal.

Page 3

What would I have to do?

If you decide you want to participate, here's what you will have to do:

1. **First you'll complete a web survey** of questionnaires about your relationship, health, and well-being. This survey should take about 30 to 45 minutes.

2. **Next, you'll fill out a web survey every day for 2 weeks.** This daily survey will only take about 5 minutes and the questions will be about experiences you have each day. You will need to fill out the daily surveys each morning. We'll send you a link each day and have the surveys set up so you can fill them out on any device where you can access the web. You can use your own personal computer, tablet, or smartphone – whatever's easiest for you.

Page 4

What else should I know?

There are a few other things we want you to know:

1. We'll pay you for filling out the surveys. We're going to pay you and your partner separately and you can each get up to $77 at the end of the 2-week study. You'll get $25 for filling out that first longer survey. Then for each daily survey you fill out we'll give you $3, for a total of $42. We really want you to fill out as many of those as you can, so if you fill out at least 12 of the 14 surveys, we'll give you a $10 bonus.

2. **If you start the study, we really want you to finish it.** If you aren't sure you have the time to participate right now, we understand. If you can't participate now but might want to in the future, just let us know!

Page 5

What's in it for me?

It's possible there will be no direct benefit to you for participating. But you might find the study interesting and learn something about yourself or your relationship by taking part in this research.

We think the biggest benefit to participating is that you have a chance to share your experiences as a sexual minority woman. Taking part in this study will help us as researchers to better understand your daily experiences, and this information could ultimately help other women.

What are the risks?

There are some risks to participating that we want to make sure you know about. In the surveys we are going to ask questions that could make you feel upset. This doesn't usually happen, but if you do feel upset and want to stop the surveys, you can. You can also call or email us and we can connect you with resources to help.

Something else we want you to know is that we'll keep the information you tell us in this study confidential. We won't share ANY of your answers to surveys with your partner or anyone else really. In fact, when you start the study we'll assign an ID number to you and we'll use this to track you through the study instead of using your name. We'll need to keep track of which ID numbers go with which names so that we can pay you at the end of the study, but we'll keep this information in a secure place and only our research staff will have access to it.

What else should I know about the benefits and risks?

We want your participation to be voluntary. This means it's ok to say you don't want to participate. And in fact, if you aren't sure you want to participate, or don't think you can do everything that's involved, then we would rather you not start the study right now.

There is more information about all of the benefits, risks, and other details we want you to know before you start the study in what's called the Informed Consent. We will show you the Informed Consent in a few more screens.

Page 6

What do I need to know about the surveys?

There are a few general things we want you to know about the surveys.

1. It's very important to us that we are learning about your experiences, so please do all the surveys alone. Don't do the surveys with your partner or anyone else, or share your responses with anyone.

2. We would prefer that you fill out each survey in one sitting. The first survey is the longest and we suggest you set aside at least 45 minutes to fill it out. The daily surveys are much shorter and you should be able to finish them in 5 minutes. If you do have to take a break in the middle of a survey, try to get back to it as quickly as you can.

3. Some of the questions might seem repetitive or similar to each other. Our research team selected these questions to help us better understand your relationship and health. Please read each question carefully and answer as honestly as possible.

4. Our research team is committed to conducting research that is inclusive and sensitive about the experiences of sexual minority individuals who participate in our studies, so we want to explain the reasons for including some of the questions in the surveys. We take seriously our responsibility to use bias-free language in our work. However, in order to conduct research that will ultimately benefit the sexual minority community, it's necessary to include questions that have been used in past research. Unfortunately, because some of these questions were developed several decades ago, at times they may not be ideally phrased, or may use a term that some people find offensive today. We hope you understand that this is necessary for research purposes only, and it's not meant to be disrespectful.

Page 7

We hope the information on the previous pages answered any questions you might have about Project Relate!

On the next page you will find the Informed Consent, which describes the study again for you. Please read this document and if you would like to participate, follow the instructions at the end of the Informed Consent.

If you have any questions, you can email us at relate@odu.edu.
