# Supplementary material for: Rationale and Design of a Remote Web-Based Daily Diary Study Examining Sexual Minority Stress, Relationship Factors, and Alcohol Use in Same-Sex Female Couples Across the United States: Study Protocol of Project Relate
Source: JMIR Res Protoc. 2019 Feb 4;8(2):e11718. doi: 10.2196/11718 (PMC6378553; doi:10.2196/11718)
Supplement: Multimedia Appendix 2 [file resprot_v8i2e11718_app2.pdf]

Appendix B

Daily Diary Items

[Affect]

The first few questions are about your mood yesterday. Yesterday I felt: [0=not at all, 6=very much]

Yesterday I felt:
- Happy
- Bored
- Relaxed
- Sad
- Excited
- Content
- Tense
- Angry

[Body Image]

Overall yesterday, how satisfied were you with your <u>body shape and weight</u>? (0 = completely dissatisfied, 10 = completely satisfied)

Overall yesterday, how satisfied were you with your <u>overall physical appearance</u>? (0 = completely dissatisfied, 10 = completely satisfied)

[Daily Relationship Functioning]

The next series of questions are about your relationship with your partner.

Yesterday, how <u>close</u> did you feel to your partner? [0=not at all, 6=very much]

Yesterday, how <u>satisfied</u> were you with your relationship with your partner? [0=not at all, 6=very much]

Yesterday, how <u>committed</u> were you to your relationship with your partner? [0=not at all, 6=very much]

Yesterday, how <u>sexually satisfied</u> were you with your relationship? [0=not at all, 6=very much]

Yesterday, how much <u>conflict</u> did you experience in your relationship? [0=none at all, 6=very much or a lot]

Yesterday, how much <u>intimacy and connectedness</u> did you feel with your partner? [0=none at all, 6=very much or a lot]

Did you see, talk to, or interact with your partner at all yesterday? [yes/no]

If no, did NOT see/talk to/interact with partner yesterday:

The following questions are about your social interactions **yesterday**. An “interaction” is talking or communicating with someone in person, by phone (voice or text messaging), or online (voice/video, messaging, on social media sites, emailing, etc.).

Mark all of the different types of people who you interacted with **yesterday**.

Spouse/partner	Stranger(s)
Parent(s)	Boss/supervisor(s)
Sibling(s)	Coworker(s)
Child(ren)	Healthcare provider(s)
Other family member(s)	Religious figure(s)
Friend(s)	Other
Acquaintance(s)	I did not interact with anyone yesterday

If any of the above EXCEPT “I did not interact with anyone yesterday”:

Now think about the most important or most meaningful interaction you had **yesterday**.

Who was this interaction with? (select one)

Parent(s)	Stranger(s)
Sibling(s)	Boss/supervisor(s)
Child(ren)	Coworker(s)
Other family member(s)	Healthcare provider(s)
Friend(s)	Other
Acquaintance(s)	

Overall, how important are the **people or person** to you? [0=not at all, 6=very much]
How important or meaningful was **this interaction** to you?
How **pleasant or positive** was this interaction?
How **unpleasant or negative** was this interaction?

Yesterday did you:

- Visit a friend or family member? [yes/no]
- Get visited by a friend or family member?
- Help someone (e.g., give someone a ride, take care of someone, give someone useful information)?
- Discuss a personal matter with someone?
- Chat by phone with someone?
- Go somewhere with someone (e.g., for a walk, shopping, to a movie, restaurant, church)?
- Ask someone for advice?
- Borrow something from someone?

If yes, did see/talk to/interact with partner yesterday in question above:

The next questions are about your interaction(s) **with your partner yesterday**.

How well do each of the following behaviors describe **your behavior yesterday** with your partner? [1 = not at all characteristic,9 = completely characteristic]

Kind or affectionate

responsive to her needs ____
patient
critical or judgmental
distant
jealous

How well do each of the following behaviors describe **your partner's** **behavior yesterday** with you? [1 = not at all characteristic, 9 = completely characteristic]

Kind or affectionate
responsive to your needs
patient
critical or judgmental
distant
jealous

At any time yesterday, did you and your partner have a conflict, argument, or disagreement, whether major or minor? [yes/no]

If yes, did have a conflict, argument, or disagreement with partner:

During the conflict, argument, or disagreement yesterday, did **you**: (mark all that apply)

Yell
Try to work out a solution
Sulk or withdraw from my partner
Throw things
Push, grab or hit my partner
Kick or hit something (e.g., the wall, door)
Make threats
Insult or call my partner names
Other:____________________________
None of the above

During the conflict, argument, or disagreement yesterday, did **your partner**: (mark all that apply)

Yell
Try to work out a solution
Sulk or withdraw from me
Throw things
Push, grab or hit me
Kick or hit something (e.g., the wall, door)
Make threats
Insult or call me names
Other:____________________________
None of the above

If no, did not have a conflict, argument, or disagreement with partner:

At any time yesterday, did you have a conflict, argument, or disagreement, whether major or minor, with any of the following people? (mark all that apply)

Friend(s)

Acquaintance(s)
Parent(s)
Sibling(s)
Child(ren)
Other family member(s)
Coworker(s)
Boss or supervisor(s)
Other(s)
None of the above

[General Stress and Coping]

The next several questions are about stressful or unpleasant experiences you may have had yesterday.

Below is a list of some common stressful or unpleasant daily experiences. Which of these happened to you **yesterday**? (mark all that apply)

Argument/conflict/disagreement
Work/school related problem
Home-related problem
Health problem or accident
Financial problem
Problem that happened to others
Other
None of the above (see follow-up below if select None here)

If "None":

Why do you think nothing stressful or unpleasant happened to you **yesterday**? (mark all that apply)

Stressful things don't usually happen to me
I avoided stressful situations
I handled situations before they became stressful
I was lucky
Another reason (free text if selected)

Do you anticipate any stressful experiences today? [yes/no]

For any of the above except for "None":

Of the events you selected, now think about the MOST stressful or unpleasant experience you had **yesterday**. Which of the following best describes this event? (mark one)

Argument/conflict/disagreement
Work/school related problem
Home-related problem
Health problem or accident
Financial problem
Problem that happened to others
Other (specify other stressor)

How stressful or unpleasant was this experience for you when it happened? [0=not at all, 6=extremely]

[Daily Sexual Minority Stressors]

Did any <u>positive</u> events occur yesterday that were <u>related to your being a sexual minority</u> or to sexual orientation issues? [Yes, No]

Did any <u>negative</u> events occur yesterday that were <u>related to your being a sexual minority</u> or to sexual orientation issues? [Yes, No]

For each of the following statements rate how much this experience describes something that happened to you **<u>yesterday</u>** **<u>because you identify as a sexual minority woman.</u>** [0=not at all, 6=very much]

I was verbally harassed by someone.

I was told I was overreacting or being oversensitive regarding sexual minority issues.

Someone responded defensively or disagreed with me when I pointed out heterosexist language or thought something was homophobic.

I heard others make fun of, mock, or call sexual minority people names.

Someone laughed at me, made jokes at my expense, or called me a name.

I was explicitly threatened with harm as a result of my sexual minority identity.

I heard anti-LGBT talk.

I perceived a situation, individual, or environment to be unsafe because of my sexual minority identity.

In thinking about your experiences **<u>yesterday</u>**, were you rejected or treated unfairly by any of the following people **<u>because you identify as a sexual minority woman.</u>** (mark all that apply)

Partner/spouse
Family member(s)
Acquaintance(s)
Friend(s)
Stranger(s)
Co-worker, colleague, or peer(s)
People in service jobs
People in helping jobs
Boss or supervisor(s)
Teacher/professor(s)
Other(s)
None of the above

<u>Yesterday</u>, did you avoid talking about topics related to or otherwise indicating your sexual orientation/identity with: (mark all that apply)

Member of your immediate family (parents and siblings)
Members of your extended family (aunts, uncles, grandparents, cousins)
People you socialize with (friends, acquaintances)
People at your work/school (coworkers, supervisors, instructors, students)
Strangers (someone you have casual conversation while in line at the store)

Other
I did not avoid talking about topics related to my sexual orientation/identity with anyone

[Drug Use]

The next several questions are about your health behaviors **yesterday**.

Did you smoke cigarettes or tobacco (including e-cigarettes) **yesterday**? [yes/no]

If No: How typical is it for you to not smoke on a day like **yesterday**? (0=not at all typical, 6=very typical)

If Yes: How many cigarettes (including e-cigarettes) did you smoke **yesterday**? ____

Did you use marijuana **yesterday**? [yes/no]

If No: How typical is it for you to not use marijuana on a day like **yesterday**? (0=not at all typical, 6=very typical)

If Yes: The next question asks about how many joints you smoked. If you use a pipe or blunt, how many average-sized joints could you have rolled? If you ingested in a different way, please indicate the equivalent number of joints. Please refer to the pictures below as examples of average-sized joints.

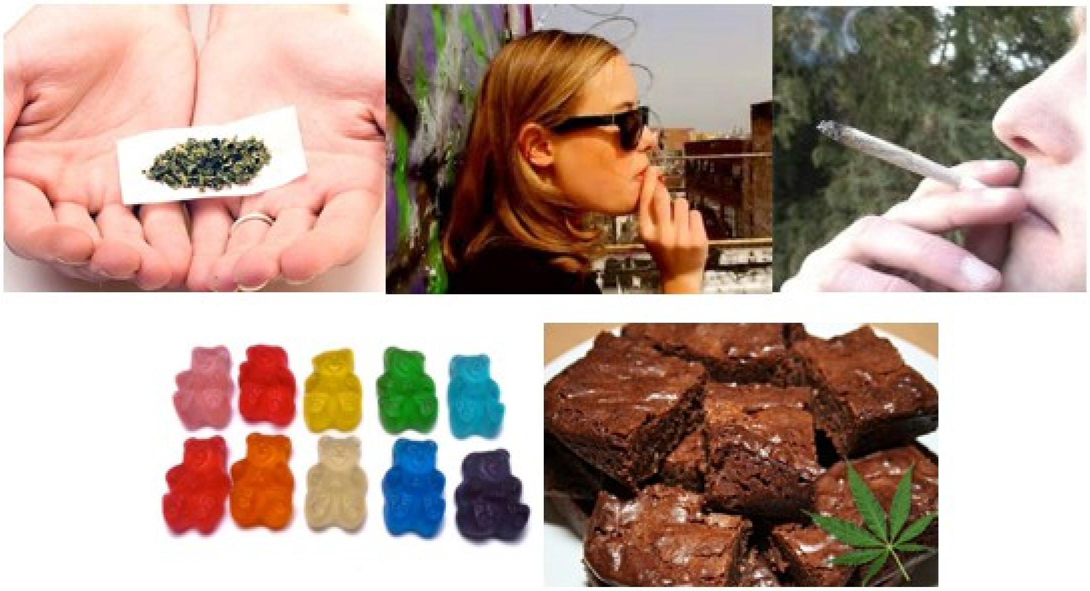

How many joints (or their equivalent) did you smoke **yesterday**? ____

[Daily Alcohol Use]

Did you drink alcohol **yesterday**? [yes/no]

If yes: The next question asks how many standard alcoholic drinks you drank. One standard drink is equivalent to 12 oz beer OR 5 oz wine OR 1.5 oz shot of liquor straight or in a mixed drink.

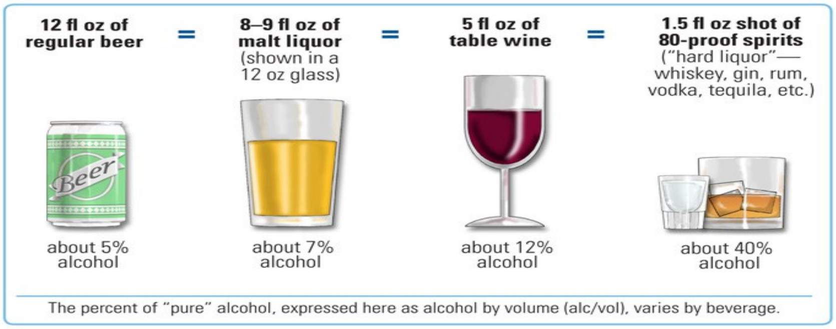

How many standard drinks did you consume **yesterday**? (drop down menu: 1 – 20+)

How many hours did you spend consuming alcohol **yesterday**? ______

On a scale from 0 to 100, how drunk did you get? (0 = not drunk at all, 100 = extremely drunk)

Who was present when you were drinking? (mark all that apply).

- Partner/spouse
- Friend(s)
- Family member(s)
- Roommate(s)
- Classmate(s)
- Professor(s)
- Coworker(s)
- Acquaintance(s)
- Stranger(s)
- Other(s)
- I was alone

If selected partner/spouse:

Was your partner:

- Present and drinking
- Present and not drinking

If select any options other than partner/spouse: no additional follow-up questions for this branch, continue to next question

Were you interacting with other people when you were drinking? [yes/no]

If yes: Of the people you were with, how many of them were also drinking?

- All of them
- Most of them
- About half of them
- Less than half of them
- None of them

<u>If no:</u> no additional follow-up questions for this branch, continue to next question

Where did you drink yesterday? (mark all that apply)

- Home
- Someone else's home
- A gay/lesbian bar or club
- A general bar or club
- A restaurant
- Another location

Below is a list of things that sometimes happen to people either during, or after they have been drinking alcohol. Indicate whether that item describes something that has happened to you as a result of **<u>yesterday's</u>** drinking.

As a result of **<u>yesterday's</u>** drinking… [Yes/No]

While drinking, I said or did embarrassing things.

The quality of my work or school work suffered because of my drinking.

I felt badly about myself because of my drinking.

I had a hangover (headache, sick stomach) the morning after I had been drinking.

I took foolish risks when I was drinking.

I felt very sick to my stomach or threw up after drinking.

I spent too much time drinking.

I neglected my obligations to family, work, or school because of drinking.

I ended up drinking when I had planned not to drink.

When drinking, I did impulsive things that I regretted later.

I found it difficult to limit how much I drank.

I had less energy or felt tired because of my drinking.

Why did you drink **<u>yesterday</u>**? [0=not at all why, 1=a little bit, 2=somewhat, 3=quite a bit, 4=definitely]

To make a gathering or party more fun or enjoyable, to celebrate a special occasion, or to be more sociable

To forget my worries or problems

Because I liked the feeling, because it was exciting, because it was pleasant or fun, or to get high

To feel less depressed or nervous

Because my friends pressured me to drink, so others wouldn't kid me about not drinking, to fit in, to be liked, or so I wouldn't feel left out

To cheer up because I was in a bad mood

To feel more self-confident and sure of myself

If did not drink yesterday:

How typical is it for you to not drink alcohol on a day like yesterday? [0=not all typical, 6=very typical]

Did you see anyone drinking alcohol yesterday? [yes/no]

Do you think any of your friends drank alcohol yesterday? [yes/no]

Why did you not drink **yesterday**? (check all that apply)

I never drink.
I only drink on special occasions.
I only drink on certain days of the week (e.g., on the weekend).
I did not feel well physically from drinking earlier this week.
I did not feel well physically for other reasons.
I experienced negative consequences the last time I drank.
I had to work at my job
I had too much school work to do
I had nobody to drink with
I couldn't obtain alcohol
I had no desire to drink
I usually don't drink on this night of the week

*[**see note below for additional questions in this branch (i.e., on non-drinking days), but these questions should appear **after** the participant completes the Drinking Intentions and Partner Drinking questions below.]*

What is the likelihood that you will drink in the next 24 hours? [slider scale, 0 = I definitely will not drink, 100 = I definitely will drink]

Did your partner drink **yesterday**? [yes/no]

If yes:

How many standard alcoholic drinks do you think your partner had yesterday? [0,1,2,3,4,…,30 dropdown]

Over how many hours do you think your partner drank? ____

How acceptable do you consider your partner's drinking yesterday to be? [1 = Strongly Unacceptable, 2 = Moderately Unacceptable, 3 = Mildly Unacceptable, 4 = Mildly Acceptable, 5 = Moderately Acceptable, 6 = Strongly Acceptable]

<u>If no:</u>

How typical is it for your partner to <u>not</u> drink alcohol on a day like yesterday? [0=not all typical, 6=very typical]

Do you think your partner will drink **<u>today</u>**? [yes/no]

*[**If did not drink yesterday, include the following questions below. If did drink, do not include these questions. This section adds an additional 18 filler items for days when no drinking is reported.]*

[Media Use – NON-DRINKING DAYS ONLY]

The next several questions are about other activities you may have done yesterday.

Yesterday, did you watch television? [yes/no]

<u>If yes:</u>

Approximately how long did you spend watching television **<u>yesterday</u>**?
[Less than 1 hour, 1 to <2 hours, 2 to <3 hours, 3 to <4 hours, 4 to <5 hours, 5 to <6 hours, 6 or more hours]

Which type(s) of program(s) did you watch yesterday? (select all that apply)
[action, animation, comedy, drama, reality TV, recreation/sports, sitcom, soap operas, talk show, other]

<u>If no:</u>

How typical is it for you to not watch television on a day like **<u>yesterday</u>**? [0=not all typical, 6=very typical]

Do you think you will watch television **<u>today</u>**? [yes/no]

Yesterday, did you use the internet? [yes/no]

<u>If yes:</u>

Approximately how long did you spend using the internet **<u>yesterday</u>**?
[Less than 1 hour, 1 to <2 hours, 2 to <3 hours, 3 to <4 hours, 4 to <5 hours, 5 to <6 hours, 6 or more hours]

What was the purpose(s) of using the internet **<u>yesterday</u>**? (check all that apply)

Check email, use social network site, view videos or pictures, shopping, read gossip site, read or post blog, read news site, play internet game, search for information, other

If no:

How typical is it for you to use the internet on a day like **yesterday**? [0=not all typical, 6=very typical]

Do you think you will use the internet **today**? [yes/no]

Yesterday, did you visit or post on a social media site? [yes/no]

If yes:

Which social media site(s) did you use (select all that apply):
[Facebook, Pinterest, Instagram, LinkedIn, Twitter, Other]

Approximately how long did you spend reading or posting on social media sites **yesterday**?
[Less than 1 hour, 1 to <2 hours, 2 to <3 hours, 3 to <4 hours, 4 to <5 hours, 5 to <6 hours, 6 or more hours]

If no:

How typical is it for you to not use a social media site on a day like **yesterday**? [0=not all typical, 6=very typical]

Do you think you will use a social media site **today**? [yes/no]

[Time Management – NON-DRINKING DAYS ONLY]

Yesterday, did you: [yes/no for each]

Make a list of the things you had to do?

Plan your day before you started it?

Make a schedule of activities for the day?

Spend time planning?

Set and honor priorities?

Find yourself doing things that interfered with your current task simply because you hate to say "no" to people?

Feel as though you were in charge of your own time?

Think about ways you could improve the way you manage your time?

Make constructive use of your time?

[Eating]

The next set of questions is about your eating yesterday. **Yesterday** when you ate, how much did you: [0=Not at all, 6=Very much]

Try to limit the amount of food you ate even when you were hungry?

Feel guilty after eating?

Lose control over your eating?

Enjoy the food you were eating?

Eat an unusually large amount of food at one time?

Try to follow strict rules about your eating (e.g., calorie limit, rules about what or when to eat)?

Eat because you were feeling stressed, sad, or upset?

[Physical Activity]

The next few questions ask you to estimate how much time you spent doing various physical activities.

How much time did you spend **sitting** yesterday? This includes time spent at work, at home, and during leisure time. This may include time spent sitting at a desk, visiting friends, reading, or sitting or lying down to watch television.

Hours: ___
Minutes: ___

Thinking only about walking that you did for at least 10 minutes at a time, how much time did you spend **walking** yesterday? This includes at work and at home, walking from place to place, and any other walking you did for recreation, sport, exercise, or leisure.

Hours: ___
Minutes: ___

Thinking only about activities that you did for at least 10 minutes at a time, how much time did you spend doing **moderate activities** yesterday? These are activities that make you breathe somewhat harder than normal like carrying light loads, bicycling at a regular pace, or doubles tennis.

Hours: ___
Minutes: ___

Thinking only about activities that you did for at least 10 minutes at a time, how much time did you spend doing vigorous activities yesterday? These are activities that make you breathe much harder than normal, like heavy lifting, digging, aerobics, or fast bicycling.

Hours: ___
Minutes: ___

[About the Survey]

These last few questions are about completing the survey today.

Where were you when you took the survey today?

Work or School
Home
Other person’s home
Other ____________

What type of device did you use to take the survey today?

Smartphone
Tablet
Laptop computer
Desktop computer
Other ___________

How private was your screen while taking this survey? [0= not at all private, 6=very private]

In order to give proper credit for completing the survey, we need to know your time zone. What is your current time zone? [eastern, central, mountain, pacific]
