# Supplementary material for: Rationale and Design of a Remote Web-Based Daily Diary Study Examining Sexual Minority Stress, Relationship Factors, and Alcohol Use in Same-Sex Female Couples Across the United States: Study Protocol of Project Relate
Source: JMIR Res Protoc. 2019 Feb 4;8(2):e11718. doi: 10.2196/11718 (PMC6378553; doi:10.2196/11718)
Supplement: Multimedia Appendix 3 [file resprot_v8i2e11718_app3.pdf]

Appendix C

End of Study Survey

We are a team of researchers who are committed to conducting research that is inclusive and sensitive regarding the experiences of sexual minority individuals who participate in our studies. However, we acknowledge that developing questions that are inclusive of all people is a difficult and ongoing task.

With that in mind, we would like to ask you a few questions about your experiences in this study, with the hope that your input may help guide us in our future research endeavors.

While answering questions related to your gender and/or sexual identity did you think that the questions you answered were inclusive of the way you describe yourself? [Yes/no]

While answering questions about your experiences as a sexual minority woman, to what degree did you think that the questions we asked captured your experience? [1=not at all, 2=a little, 3=somewhat, 4=mostly, 5=a great deal]

What ways could we be more inclusive of your sexual and/or gender identity in future studies? [open ended question]

Do you think that there were other experiences that we did not ask about, that you would have liked us to ask about? [yes/no]

> <u>If yes</u>: what kinds of experiences do you think we should consider asking future participants? [open ended question]

In this study, you completed daily surveys and we are interested in your feedback on these surveys.

The following questions are about the **<u>surveys that you completed each morning</u>**, using the link that we emailed you daily.

What type of device did you use to complete the surveys (mark all that apply):
- Smartphone
- Tablet (ipad, Kindle Fire, etc.)
- Laptop or desktop computer
- Other

What type of device did you use <u>the most</u> to complete the surveys each morning?
- Smartphone
- Tablet (ipad, Kindle Fire, etc.)
- Laptop or desktop computer
- Other

What type of smartphone did you use for this study?
- Apple (iPhone)
- Android (Samsung, LG, HTC, etc.)
- Other _____
- I did not use a smartphone for any surveys

How easy was it to access and complete the survey from the link we sent you? [0=not at all easy, 3=moderately, 6=very easy]

Would you have preferred to receive a text message with the survey link instead of an email? [yes/no]

Did you think the length of the survey you completed each morning was: [0=too short, 3=the right length, 6=too long]

Was 14 days of daily surveys…[not enough days, the right amount, too many days]

One of the purposes of this study was to gain a better understanding of sexual minority women's health and well-being. The following questions ask about your physical and mental health.

	Strongly Disagree			Neutral			Strongly Agree
I would like to improve my physical health	1	2	3	4	5	6	7
I would like to improve my mental health	1	2	3	4	5	6	7

I would be interested in improving my health in the following areas (mark all that apply):

- Reduce my drinking
- Reduce or quit smoking
- Get more physically active
- Eat healthier
- Lose weight
- Improve my sleep
- Reduce my stress
- Reduce my depression or anxiety
- Reduce my use of substances other than alcohol
- Other _______

The following questions ask about are about your willingness to use mobile technology to track or change aspects of your physical and mental health. By mobile technology, we mean either a smartphone, tablet, or iPad. When answering the questions, think about whichever you use most frequently or is easiest for you to use.

	Strongly Disagree			Neutral			Strongly Agree
I am willing to use mobile technology for tracking my health	1	2	3	4	5	6	7
I am willing to use mobile technology to help me try to change my health or behaviors	1	2	3	4	5	6	7
I think using mobile technology for healthier physical health behaviors (e.g., apps for eating habits, physical activity, drinking, etc.) can improve my physical well-being	1	2	3	4	5	6	7
I think using mobile technology for mental health improvement (e.g., apps for tracking	1	2	3	4	5	6	7

mood, relaxation, etc.) can improve my emotional well-being							
I think I would be successful in using mobile technology to improve my health	1	2	3	4	5	6	7
I think I would enjoy using mobile technology to improve my health	1	2	3	4	5	6	7
Mobile technology is easy to use for me	1	2	3	4	5	6	7
Mobile technology is affordable for me	1	2	3	4	5	6	7

Thank you for providing this valuable feedback about your participation in Project Relate! If you are interested in learning about the results of this study, please enter your email address below: ____________.

If you are interested in being considered for future research studies, please enter your email address below: ______________.
